# Impact of sequencing depth on the characterization of the microbiome and resistome

**DOI:** 10.1038/s41598-018-24280-8

**Published:** 2018-04-12

**Authors:** Rahat Zaheer, Noelle Noyes, Rodrigo Ortega Polo, Shaun R. Cook, Eric Marinier, Gary Van Domselaar, Keith E. Belk, Paul S. Morley, Tim A. McAllister

**Affiliations:** 1 Lethbridge Research and Development Centre, 5403 1 Ave South, Lethbridge, AB T1J 4P4 Canada; 2 Department of Clinical Sciences, Colorado State University, Fort Collins, CO 80523 USA; 3 Department of Biological Sciences, University of Lethbridge, 4401 University Drive, Lethbridge, AB T1K 3M4 Canada; 4 Alberta Agriculture and Forestry, 100, 5401–1st Avenue South, Lethbridge, AB T1J 4V6 Canada; 5 National Microbiology Laboratory, Public Health Agency of Canada, 1015 Arlington Street, Winnipeg, MB R3E 3R2 Canada

## Abstract

Developments in high-throughput next generation sequencing (NGS) technology have rapidly advanced the understanding of overall microbial ecology as well as occurrence and diversity of specific genes within diverse environments. In the present study, we compared the ability of varying sequencing depths to generate meaningful information about the taxonomic structure and prevalence of antimicrobial resistance genes (ARGs) in the bovine fecal microbial community. Metagenomic sequencing was conducted on eight composite fecal samples originating from four beef cattle feedlots. Metagenomic DNA was sequenced to various depths, D1, D0.5 and D0.25, with average sample read counts of 117, 59 and 26 million, respectively. A comparative analysis of the relative abundance of reads aligning to different phyla and antimicrobial classes indicated that the relative proportions of read assignments remained fairly constant regardless of depth. However, the number of reads being assigned to ARGs as well as to microbial taxa increased significantly with increasing depth. We found a depth of D0.5 was suitable to describe the microbiome and resistome of cattle fecal samples. This study helps define a balance between cost and required sequencing depth to acquire meaningful results.

## Introduction

Over the past decade the field of metagenomics has enabled substantial advancement in the knowledge of microbial ecology, evolution, and diversity. This includes a more in-depth understanding of complex microbial communities within the gastrointestinal tract of animals and humans. Culture-independent metagenomic approaches can aid in understanding the ecological role, phylogeny, and functionality of gut microbial communities in relation to host physiology^1–4^. Metagenomic shotgun sequencing investigations have been used to reveal specific features of the resistome^5–9^, mobilome^10,11^, virulome^12^ and virome^13^. The increasing prevalence of antimicrobial resistance (AMR) in bacteria is one of the most important challenges facing public health. It has been proposed that livestock production systems may contribute to an increased prevalence of antimicrobial resistant bacteria and genes in the environment and therefore pose a risk to human health. Globally, more than 57 million kilograms of antibiotics are used annually in food animal production^14^, which may select for antibiotic-resistant bacteria that persist throughout the meat and milk production chain. Investigation of the microbiome and resistome of farm animals and their environment may provide valuable data and models to estimate the public health risk of antibiotic-resistant human infections associated with antibiotic use in food-animals.

Metagenomic shotgun sequence analyses are accomplished by unrestricted sequencing of the genomes of all microorganisms present in a sample, including uncultured organisms. Fully capturing all DNA sequences originating from every microorganism in a complex environment is impractical despite continuous improvements in sequencing depth. Estimating the required sequencing depth needed to characterize a particular microbiome is important to fulfill the objectives of a given study, such as understanding the ecology of antibiotic resistant genes and bacteria in food production systems. However, the relatively high cost of metagenomic sequencing can still limit the amount of sequence reads that can be generated, impacting the biological interpretation of resulting data^15^. Here, we investigate the effect of varying sequencing depth on the ability to gain information about the taxonomic structure and prevalence of antimicrobial resistance genes (ARGs) of the fecal microbial community from beef cattle.

## Results and Discussion

### Sample processing and DNA isolation

Sample processing and DNA isolation are the first and crucial steps in any metagenomic analysis. It is imperative to preserve the integrity of the microbial community in a sample from the time of collection until nucleic acids are extracted. Samples were placed on ice immediately after collection and flash-frozen in liquid nitrogen upon arrival in the lab, within 24 hours of collection, with subsequent storage at −80 °C to ensure preservation of sample integrity. No reliable archetype exists for the extraction of metagenomics DNA from complex microbial communities. Minimally biased nucleic acid extraction procedures are desired to generate DNA for metagenomic shotgun sequencing that accurately reflects the genomic content of the community from which it was derived. In principle, the extracted DNA should be representative of all cells present in the sample. Furthermore, adequate quantities of high-quality DNA must be obtained for subsequent library preparation and sequencing. Fresh feces may contain up to 10 billion bacterial cells per gram in addition to protozoa, fungi and viruses. Choice of DNA extraction method from fecal samples clearly influences the information gained on community structure^16^. All DNA isolation methods from feces can contribute to variation, including differences in cell wall lysis between Gram-positive and Gram-negative bacteria, sensitivity of regions of DNA to inhibitors such as humic acids and the amount of sample extracted^17^. The DNA extraction method described here was developed and optimized in our lab with the purpose of minimizing such biases and to ensure reproducibility. We used bead-beating to enhance the yield of DNA from Gram-positive bacteria and denaturants including guanidine isothyocynate and β-mercaptoethanol to shield DNA from nucleases after cell lysis. A recent study^18^ assessed DNA extraction using various methods and demonstrated that sample type and DNA isolation procedure had a significant impact on genomic inference of microbiome composition and that bead beating increased the extraction of DNA from Gram-positive bacteria. Using the extraction procedure described in present study, both the yield and the quality of DNA were highly reproducible with no indication of PCR inhibitors observed as demonstrated by successful 16S rRNA gene amplification of both undiluted and diluted versions of DNA samples (Supplementary info file; Table S1, Figures S1 and S2).

### Metagenomic DNA sequencing

**Figure 1:**
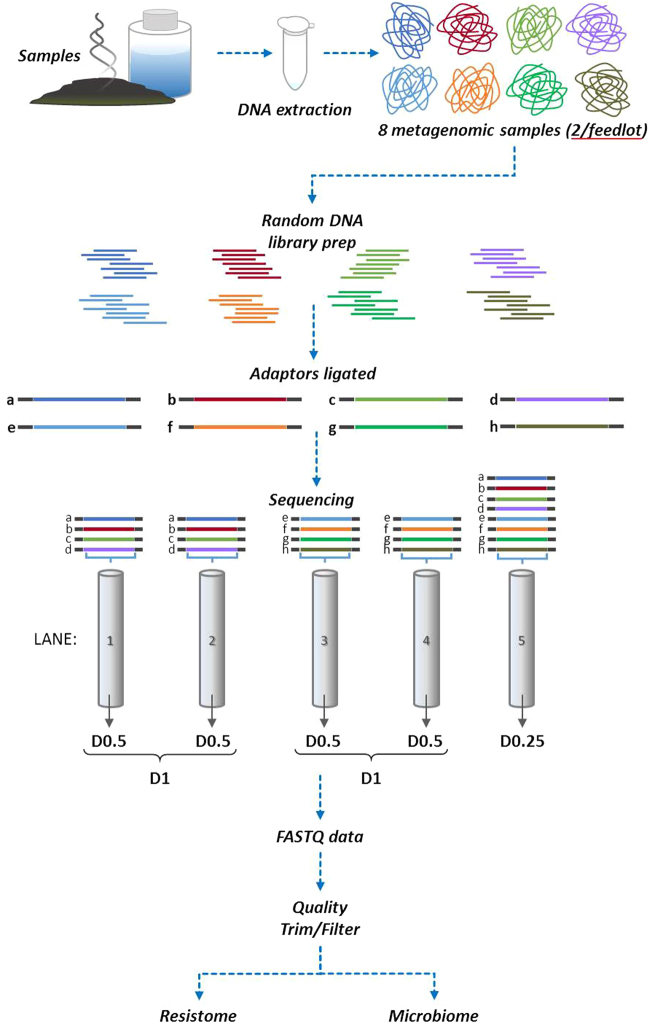
Study design and workflow for sequencing trial to estimate sequencing coverage requirements. Two individual bovine fecal samples were collected from each of the four feedlots (n = 8 total). Two sample pools were created with each pool containing 4 samples (n = 4 × 2 pools). Each pool was run in duplicate in its own sequencing lane to assess technical variation and to provide a doubling dilution (D0.5) of the original material (D1; combined 0.5 duplicates). An additional pool was created containing genomic libraries from all eight samples to create an additional doubling dilution (D0.25) relative to the D0.5 sample pools.

Processing of Illumina HiSeq. 2000 metagenomic sequencing data at various sequencing depths (Fig. 1) produced 940 million reads for D1, 470 million reads for each of the two D0.5 replicate sets and 204 million reads for D0.25 across all 8 samples with average values of sample read counts of 117, 59 and 26 million, respectively (Supplementary Dataset 1). The average read quality score for samples ranged from 33 to 37. Of the total reads generated, 94–97% survived quality filtering and trimming across all datasets. Considering that the fecal samples originated from cattle, we evaluated the level of bovine DNA in our samples using the BWA reference mapping tool with default parameters^19^ which matched reads to the *Bos taurus* reference genome (UMD_3.1.1). Of the total quality-filtered reads, on average 0.27% reads were associated with the host genome (range 0.06–0.96%) across all datasets. Other studies have also demonstrated similar levels of host genetic material in metagenomic sequence obtained from bovine fecal samples^6,7^. The host genome filtering was performed as a stand-alone process and not as a part of the metagenome-resistome analysis workflow. For samples with high potential of host genome as a major contaminant (e.g., carcass sponge and trimming samples^7^), host read removal prior to taxonomy and ARG assignment could be useful in reducing the size of the dataset.

### Microbiome comparison at various sequencing depths

Of a total of 2.1 billion reads across all datasets, 46.5 million reads (2.23%) were identified at the bacterial and archaeal phyla level using Kraken^20^ with the custom Kraken database comprised of complete genomes in RefSeq for bacteria, viruses, fungi, protozoa and archaea (bvfpa). The taxonomic assignments in a metagenomics study are heavily dependent on computational tools to extract reliable information about the community in question. Of the number of tools available to investigate the taxonomic composition of metagenomes, Kraken has been ranked among the fastest with high-accuracy classification^21^ and with an ability to map over 70% of the provided reads^21,22^. The large proportion of uncharacterized (>97%) reads in this study may be a reflection of the presence of eukaryotic (feed-associated plants) DNA in the metagenomics samples considering that plant genomes were not part of the bvfpa database. For metagenomic sequence data analyzed in present study, between 1% to 4.5% of the Kraken-assigned reads were identified as belonging to bacteriophage PhiX174 genome and these were filtered out from Kraken output data prior to downstream analysis. PhiX174 *sensu lato* virus genomic DNA is used as a quality and calibration control for Illumina sequencing runs; it is generally spiked in the same lane along with the sample. During post-sequencing process of de-multiplexing (binning sequence reads into separate files for each index tag), some unintended binning of PhiX174 reads can occur primarily from index misassignment due to image registration errors. Consequently, PhiX DNA has been identified as a major contaminant of NGS data, a reflection of a lapse in either the application or effectiveness of proper quality control measures^23^. Use of PhiX174 DNA as a sequencing control may therefore require subsequent quality control steps such as mapping demultiplexed reads against the PhiX174 genome to filter the sequences corresponding to this bacteriophage so that they are absent in the final read data. This would be especially relevant if the sequence data is to be used for a genomic/metagenomic assembly. Recent studies have revealed that of the >1000 genomes in public databases (i.e. Genbank), ~10% are contaminated with PhiX174 sequences^23^.

**Figure 2:**
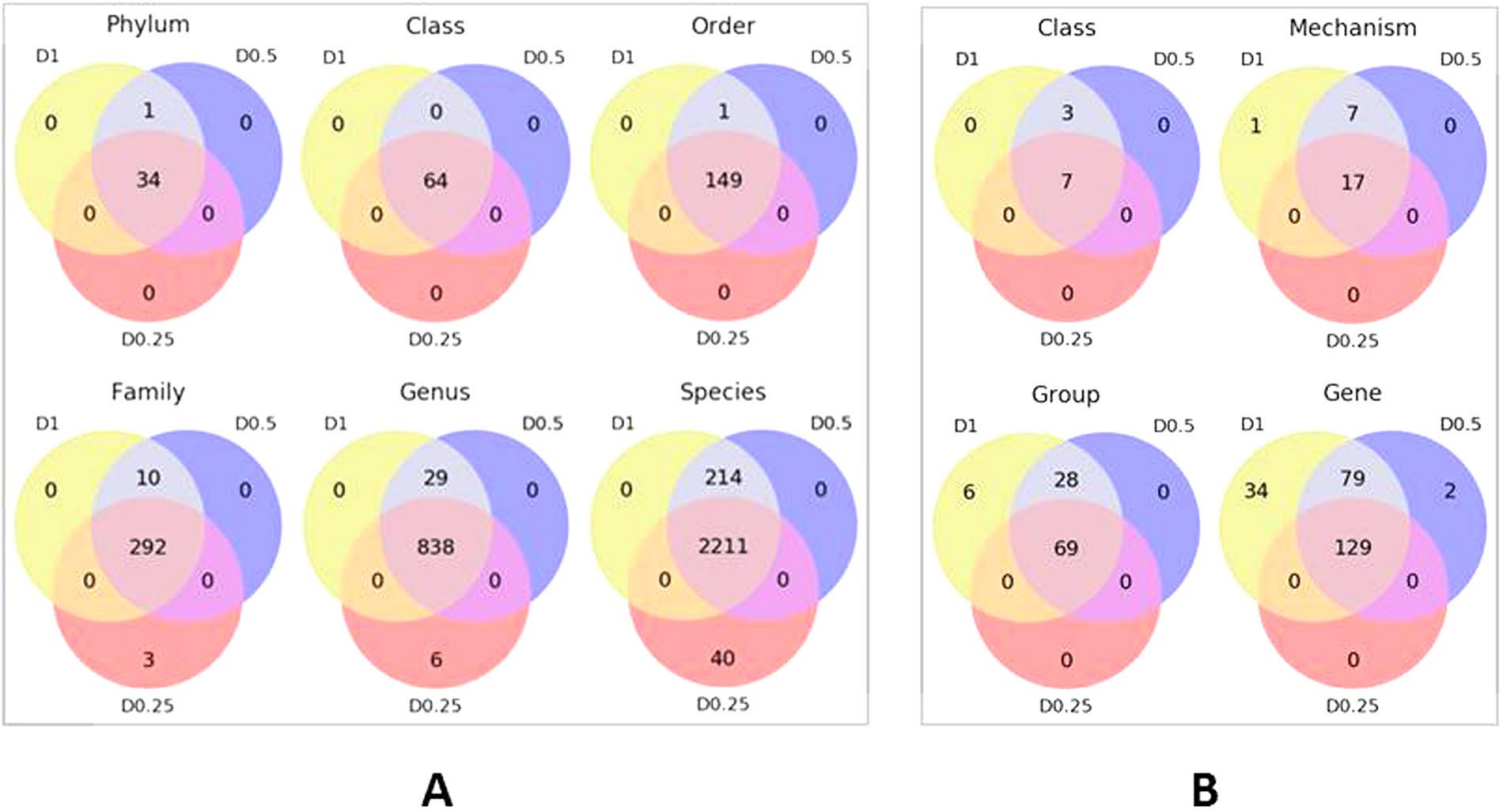
Venn diagrams representing, (**A**) the intersection of various microbiome taxonomic levels between datasets obtained at D1, D0.5 and D0.25 sequencing depths, and (**B**) the intersection of various classification levels of the resistome between datasets obtained at D1, D0.5 and D0.25 sequencing depths.

Across all datasets associated with samples sequenced at D1, D0.5 and D0.25 sequencing depth (Fig. 1), significant correlations (Spearman’s rho = 0.93) were observed between the number of reads and the number of Kraken hits at the Phylum level (Figure S3A). The Kraken assignment values for duplicate datasets in D0.5 depth group were comparable assuring consistency of replicates. The average number of reads aligning to phyla increased by ~2.4 fold for D0.5 as compared to D0.25, and by 2 fold for D1 as compared to D0.5 (Supplementary info file; Figure S4). For D1 and D0.5 datasets reads were assigned to the same 35 bacterial and archaeal phyla; whereas 34 of 35 phyla were identified at D0.25 (Fig. 2A; Supplementary Dataset 2). Similarly, 64 classes, 149 orders, 292 families, 838 genera and 2,210 species were shared between all three depths (Fig. 2A) and new taxa were increasingly identified with greater sequencing depth. At lower taxonomic levels including family, genus and species, more taxa were discovered at D1 and D0.5 than D0.25 (Fig. 2A). Those differentially present taxa had a very low abundance (1–6 reads) in corresponding samples and although some were comprised of bacteria and archaea, the majority were related to bacteriophages. This indicates sporadic sequence capture of low abundance taxa as well as a low prevalence of temperate and/or lytic bacteriophages associated with the bacterial and archaeal genomes present in the bovine fecal microflora. Although the number of studies investigating the microbiome has proliferated with the advent of NGS, the majority of studies of the microbial community have been based on amplicon sequencing of regions of 16S rRNA gene rather than shotgun metagenomics^24^. As a result they do not provide information on the prevalence or diversity of the virome. Our study indicates that to capture the profile of low-abundance organisms (e.g., viruses) in similar environmental samples, deeper sequencing is required.

**Figure 3:**
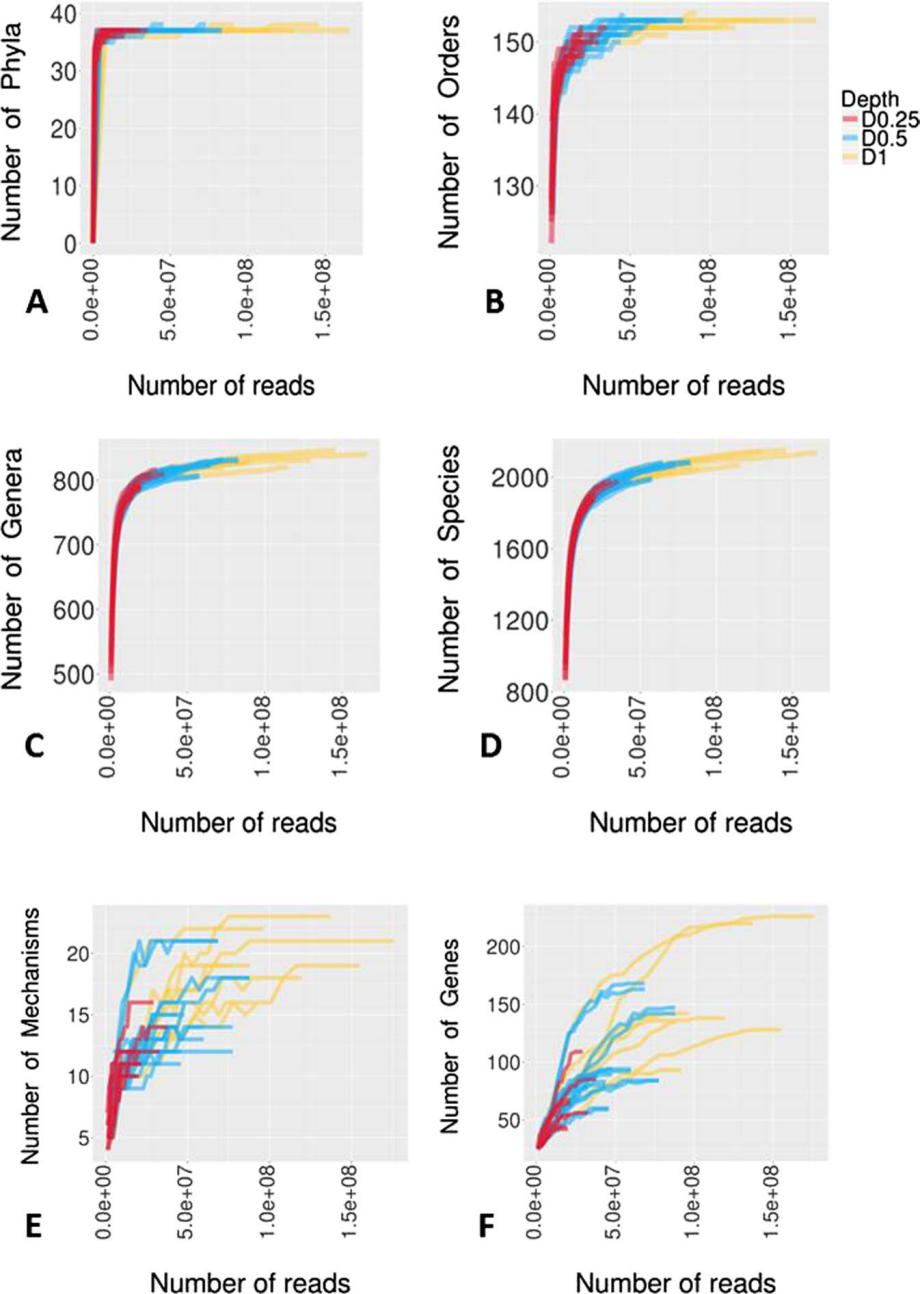
Comparison of microbiome (**A**–**D**) and resistome (**E**,**F**) richness and coverage at different taxon levels in three metagenomic data sets at sequencing depths of D1, D0.5 and D0.25 for all 8 samples using rarefaction curves.

Rarefaction curves were used as a qualitative method to estimate the species richness as a function of sequencing depth at the various taxonomic levels (Fig. 3A–D; Supplementary Dataset 3). For both D1 and D0.5, rarefaction curves reached their asymptotes or started to plateau for all taxa levels, suggesting that saturation in sequencing was achieved. However, for D0.25, only rarefaction curves associated with higher taxa plateaued, indicating that D0.25 was insufficient to describe the true species richness in metagenomic samples.

**Figure 4:**
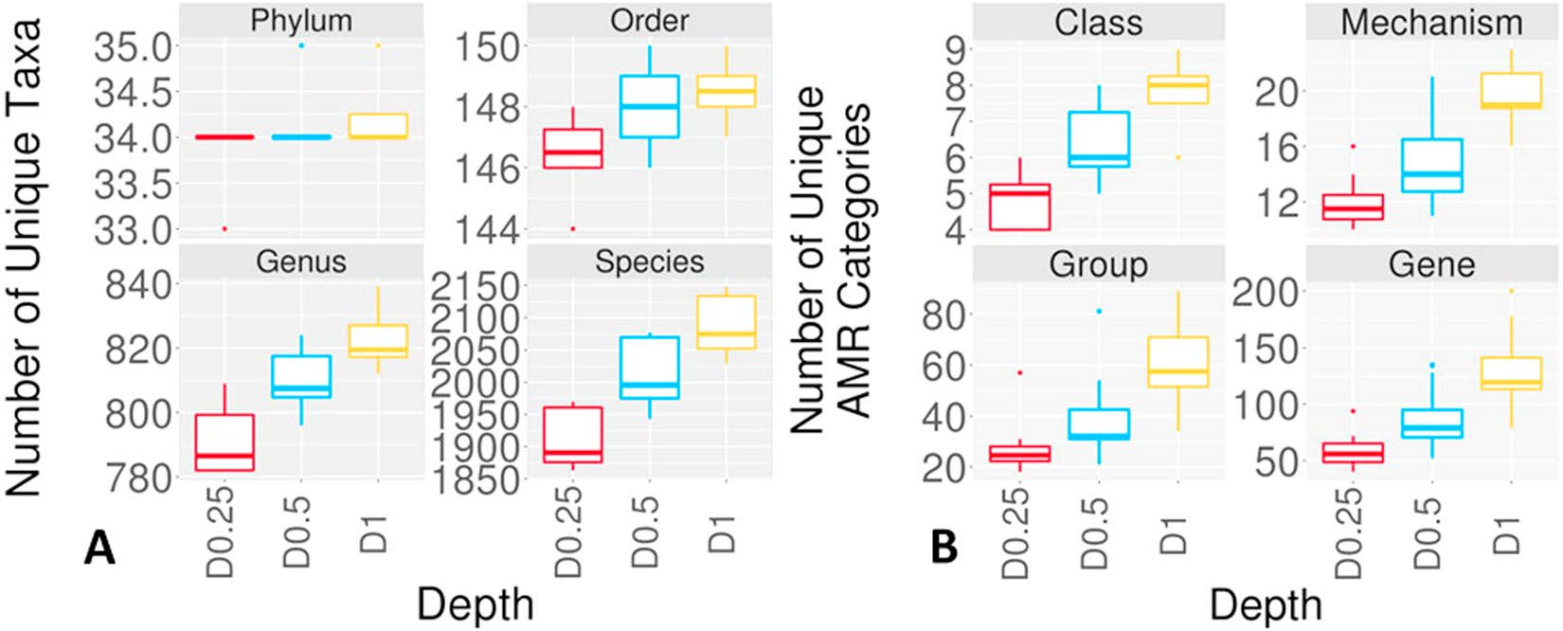
Richness of microbiome and resistome at various sequencing depths. Box-and-whisker plots showing (**A**) microbial taxon richness, and (**B**) AMR category richness. Boxes represent the interquartile ranges (upper line is the 75% quantile, and the lower line is the 25% quantile), the lines inside the boxes are the medians, the whiskers span the range of the 25% quantile or the 75% quantile plus 1.5 times the interquartile range, and dots are outliers.

Richness of the fecal microbiome did not differ at the phyla (p = 0.15) or class (p = 0.20) levels across all three sequencing depths (Fig. 4A). However, richness increased with increasing sequencing depth for lower taxa at the order (p = 0.013), family (p = 0.001), genus (p = 0.001), and species (p < 0.001) levels. With post hoc analyses, richness was higher for D1 than D0.25 at the order (p = 0.017), family (p = 0.001), genus (p < 0.001), and species levels (p < 0.001). Furthermore, richness was higher for D0.5 than D0.25 at the genus (p = 0.024), and species (p = 0.016) levels. However, richness of the orders (p = 0.075), and families (p = 0.10) did not differ between these two sequencing depths.

**Figure 5:**
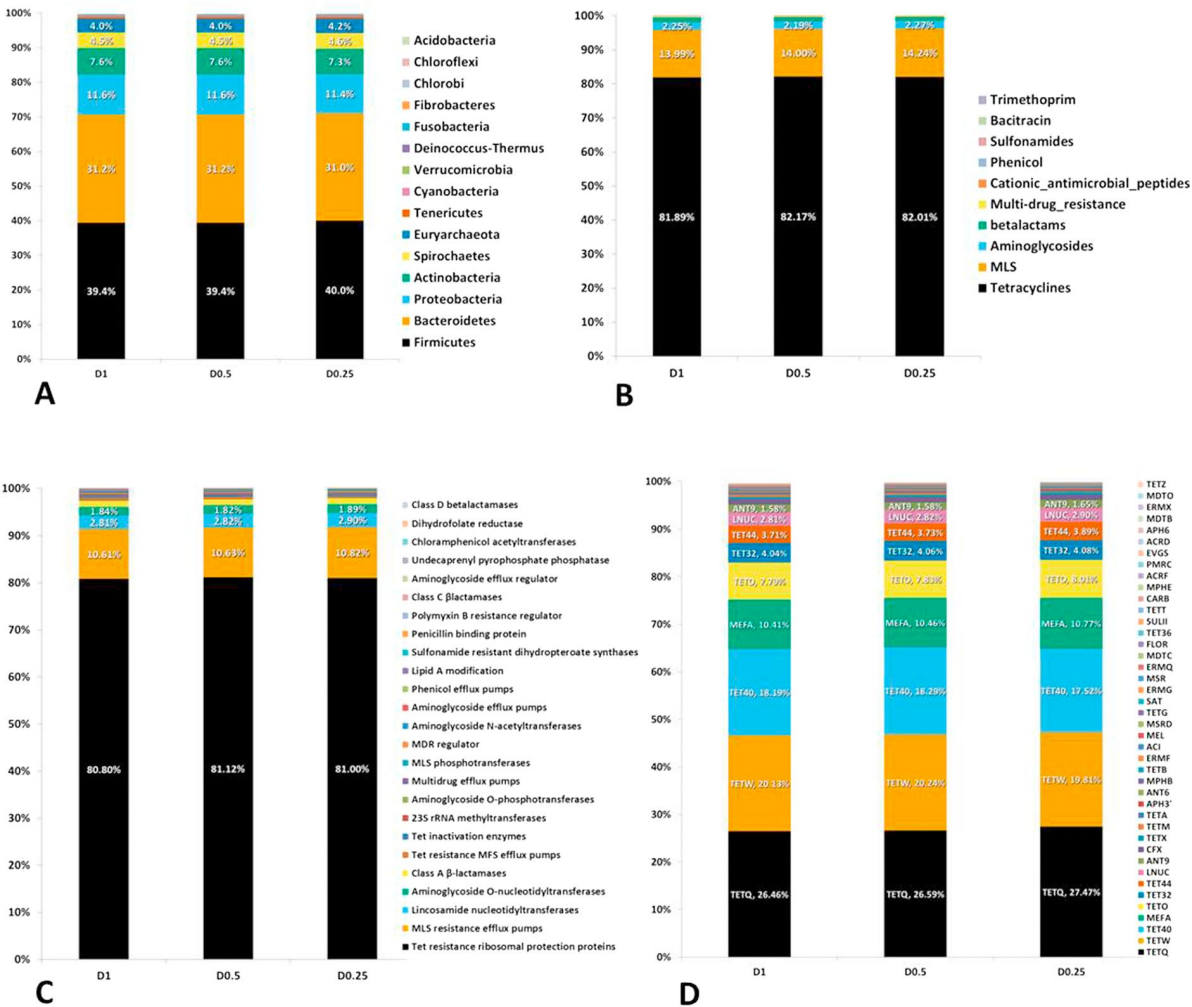
Relative abundance of microbial taxa and AMR annotation levels. Microbial phyla (**A**), AMR classes (**B**), AMR mechanisms (**C**) and AMR gene groups (**D**) across various sequencing depths of D1, D0.5 and D0.25.

The α-diversities of the taxonomic levels analyzed (p = 0.98–1.0) did not differ among sequencing depths as indicated by the distributions of the Inverse Simpson’s and Shannon’s index (Figures S5A and C). Regardless of sequencing depth, the relative proportions of reads assigned to the major bacterial phyla Firmicutes, Bacteroidetes, Proteobacteria and Spirochaetes remained similar (approximately 40%, 31%, 11.5, 7.5% and 4.5% respectively; Fig. 5A). For the archaeal phylum Euryarchaeota, the proportion of reads across all sequencing depths remained ~4.5% with methanobacteria as the most prevalent class in this phylum. Others have found that increasing the sequencing depth more consistently estimated the relative abundance of microbial taxa^25^. However, those findings may reflect the limited sequencing depth of that study which ranged from 500–100,000 sequences across samples as compared to the 19–175 million sequences obtained in our study across three levels of depth (Supplementary Dataset 1). Compared to our study, a rumen microbial metagenome study^26^ using 43.4–72.7 million 100 bp PE reads (similar to D0.5 range in our study) identified similar compositions for the top three abundant phyla; Firmicutes, Bacteroidetes and Proteobacteria. Interestingly, unlike using complete genomes from the RefSeq database for taxonomic assignments as in our study, their taxonomic assignments were based on bacterial phyla reads mapping to 16S rRNA gene sequences in the Greengenes database. Although species richness increased with sequencing depth, based on rarefaction and richness analyses combined, sequence reads resulting from the minimum sequencing depth in this study (D0.25) were adequate to infer the structure and relative abundance of members of the microbial community at the order level.

### Resistome comparison at various sequencing depths

Approximately 5.2 million reads (0.25% of total reads) were found to be associated with the MEGARes AMR database across all datasets. We selected a ≥75% gene fraction threshold (100% nucleotide homology with reference across 75% of target gene) for reads to be considered as ARG-associated hits unlike previous studies which used an 80% gene fraction threshold^6,7,27^. This approach resolved inconsistent filtering of ARGs across datasets with a gene fraction threshold difference of as low as 0.02. For example, read assignment to aminoglycoside AMR group Ant9 determinants was inconsistent between duplicate D0.5 datasets and between various depths for the same sample with coverage ranging between 78–90% with elimination of corresponding reads below the 80% threshold.

As expected, increasing the sequencing depth increased the number of AMR determinants identified (Spearman’s rho = 0.99; Figure S3B). Similar to Kraken assignment data, the ARG assignment values for duplicate datasets in D0.5 depth group were comparable. The number of reads aligning to the MEGARes ARGs database increased 2.4 fold for D0.5 versus D0.25, and by 2 fold for D1 versus D0.5 (Supplementary info file; Figure S3). A total of ~2.4 million reads from D1 (n = 8) and ~2.4 million reads from D0.5 (~1.2 million reads per replicate set where n = 2 × 8), constituting about 0.25% of total sequence reads for each depth group, were assigned to the same 10 AMR classes. These included tetracyclines, macrolides, aminoglycosides, β-lactams, multi-drug resistance efflux pumps, cationic antimicrobial peptides, phenicols, sulfonamides, bacitracin and trimethoprim. For D0.25, ~0.5 million ARGs associated reads (~0.24% of total reads) corresponded to 7 of the 10 AMR classes identified at D1 and D0.5 (Fig. 2B; Supplementary Dataset 4). The undetected AMR classes in D0.25 included phenicol, bacitracin and trimethoprim. The low ARG prevalence for these AMR classes may be a reflection of the fact that these antimicrobials were not being used to any appreciable extent in the cattle from which the fecal samples were collected. An association between a specific antimicrobial use and related AMR has been previously observed. A significant decrease in ceftiofur-resistance was observed in *Salmonella* Heidelberg isolates from retail chicken and humans following a voluntary withdrawal of ceftiofur for disease prophylaxis in hatcheries^28^. A more recent study^29^ also reported a lower prevalence of ARGs in beef cattle raised without antibiotics. Interestingly, all of the missing classes in D0.25 belonged to category III, a medium importance antimicrobial drug category based on importance in human medicine, as they are not the preferred option for treatment of serious human infections.

At the lower (than class) ARG annotation levels, 17 AMR mechanisms, 69 ARG groups and 129 ARG determinants were shared between all three sequencing depths (Fig. 2B). Additional ARGs were identified with increasing sequencing depth with these discoveries being more prominent when depth moved from D0.25 to D0.5 than from D0.5 to D1 (Fig. 2B). Along with 17 AMR mechanisms common to all depths, 7 additional AMR mechanisms belonging to various AMR classes were identified in both D1 and D0.5. These additional mechanisms included dihydrofolate reductases (trimethoprim), aminoglycoside efflux regulators (aminoglycosides), polymyxin B resistance regulators (cationic antimicrobial peptides), class C β-lactamases (β-lactams; category II), undecaprenyl pyrophosphate phosphatases (bacitracin), chloramphenicol acetyltransferases (phenicol) and phenicol efflux pumps (phenicol). One mechanism belonging to Class D β-lactamases was only identified in the D1 dataset. As expected, rare ARGs were more frequently discovered at higher sequencing depths and were often absent in datasets generated with a lower sequencing depth (Figure S6, Supplementary info file; Supplementary dataset 4). Depending on the degree of interest in these rare ARGs, as well as their implications for human and animal health, it may be necessary to use greater sequence depths to consistently monitor their presence.

Rarefaction curves were generated to assess the saturation of samples at each depth for various AMR categories (Fig. 3E,F; Supplementary dataset 3). For both D1 and D0.5, saturation in sequencing was achieved up to the mechanism levels, whereas D0.25 did not reach an asymptote. At the individual gene level asymptotes were never achieved for all datasets. This could be a reflection of the presence of multiple closely related variants of various ARGs in the bacterial populations also represented in the MEGARes database used to analyze the resistome. Hence, it may perhaps be more practical to focus the resistome comparisons at the mechanistic level.

The richness of AMR determinants increased with increasing sequencing depth (Fig. 4B). Compared to microbiome phyla richness, more heterogeneity was detected in the high level AMR category among samples which is a reflection of apparently significantly smaller membership size for AMR categories compared to the microbial taxa. Also, the host microbiome is expected to be more consistent than a resistome which may be impacted by antimicrobial use variation among animals. Richness differed (p = ≤0.001) at the class, mechanism, group and gene levels. Post hoc comparisons revealed that richness was higher (p = ≤0.001) for D1 than for D0.25 at all AMR taxa levels. When comparisons were made between D1 and D0.5, richness was higher for D1 at the mechanism level (p = 0.028), but not at the class (p = 0.12), group (p = 0.07), or gene (p = 0.12) level. In addition, richness of D0.5 samples did not differ significantly from D0.25 at the class (p = 0.051), mechanism (p = 0.13), group (p = 0.16), or gene level (p = 0.08). The resistome α-diversities increased with increasing sequencing depth (Figure S5B and D). However, statistical analyses revealed that the distributions of the Inverse Simpson’s and Shannon indices did not differ (p = >0.05) at all depths.

Similar to microbiome results, the relative proportion of sequences associated with different AMR determinants remained similar at all sequencing depths for abundant determinants (Fig. 5B–D). At the class level, tetracycline resistance was the most prevalent (82%) followed by macrolide, aminoglycoside and β-lactams, respectively (Fig. 5B; Supplementary Dataset 5). The tetracycline resistance ribosomal protection protein mechanism was most abundant (81%) at all sequencing depths followed by macrolide resistance efflux pumps (~11%; Fig. 5C). TetQ, TetW and Tet40 were among the most prevalent tetracycline resistance genes identified; followed by macrolide resistance efflux pump genes belonging to the MefA group (Fig. 5D). Previous studies^6,7^ also indicated a high prevalence of genes within the tetracycline resistance class, with 98% of reads aligning to ribosomal protection proteins represented in TetQ and TetW groups. These tetracycline resistance groups were also the most prevalent in fecal samples collected from humans^30,31^, suggesting their high abundance in both cattle and human populations. In addition, studies across diverse agricultural ecosystems also demonstrated the ubiquity of tetracycline resistance genes^32,33^. Among the second most prevalent resistance class macrolide in our study, the MefA group was dominant within fecal samples. Its higher relative abundance in cattle feces could be due to its common presence in enteric bacteria^34^ or due to co-selection along with other ARGs as many tetracycline ARGs are linked to macrolide ARGs through common mobile genetic elements^35^.

In North America, the use of in-feed tetracycline and macrolides to prevent liver abscesses and other bacterial diseases and to improve feed efficiency is a common management strategy in beef cattle production. Macrolides are also used to treat and manage Bovine Respiratory Disease (BRD) in cattle. A high prevalence of both tetracycline and macrolide resistance classes in bovine feces could be a reflection of ubiquitous use of these antibiotics in the beef production system, considering the reported linkage between administration of tetracycline and macrolides to cattle and increases in the abundance of relevant tetracycline and macrolide ARGs in cattle feces^36,37^.

Overall, the resistome analysis results emphasise that increasing sequencing depth is helpful in detecting rare resistance genes, particularly if those rare genes belong to important drug categories. Our data demonstrate that D0.5 with ≥50 million reads would be a suitable compromise for sequencing bovine fecal samples and adequately inferring their resistome, considering that no further classes were discovered by the D1 sequencing depth and only a single unique mechanism was discovered as compared to the D0.5 level. It has been recommended that coverage is not simply a function of data set size but also depends on the complexity of the communities sampled^38^. The majority of published metagenomic studies describe 16S rRNA gene-based community structure evaluations; whereas published shotgun-based studies do not usually determine sequencing depth requirements for described studies. To our knowledge this is the first in-depth report describing the sequencing coverage requirements for simultaneously evaluating both the microbiome and resistome. In addition to providing a snapshot of the bovine gut microbiome and resistome, the results presented here lay the groundwork for understanding the relationship between the richness and diversity of microbiome and resistome and limitations posed by sequencing coverage. Similar pilot studies are recommended for other sample types and matrices prior to undertaking a metagenomics sequencing venture involving a large number of samples.

## Methods

### Sample collection, DNA isolation, quantitation and quality assessment

Composite fecal samples (n = 8) analyzed in this study were collected from four different feedlots (2 samples each) in southern Alberta. Sampling procedures were reviewed and approved by the Lethbridge Research Centre Animal Care and Use Committee, and were carried out in accordance with the committee’s approved guidelines.

Composite fecal samples, each comprised of approximately equal portions of 20 individual fresh fecal pats from within a pen were collected, thoroughly mixed, and approximately 20 g aliquots were placed in 18 oz Whirl-Pak bags for shipment to the laboratory on ice. Upon arrival, within 24 hours of collection, sample bags were flattened to create thin sheets, flash frozen in liquid nitrogen and stored at −80 °C.

Metagenomic DNA was extracted from bovine feces as follows: frozen fecal sample (325 mg) was added to a sterile 2.0 mL safe-lock snap-cap tubes containing 0.4 g of sterile zirconia beads (0.3 g of 0.1 mm and 0.1 g of 0.5 mm). One milliliter of resuspension buffer (600 mM NaCl, 120 mM Tris-HCl, 60 mM EDTA, 200 mM guanidine isothyocynate) and 5 µL of β-mercaptoethanol (β-ME) were added to the sample tube and mixed followed by the addition of pre-heated (70 °C) 10% SDS (200 μL) and homogenization for 3 min at maximum speed on a Qiagen TissueLyser™ (setting = 30). The homogenate was then incubated at 70 °C for 15 min with shaking at 350 RPM followed by centrifugation at 4 °C for 5 min at 16,000 × g to obtain supernatant. To recover DNA from any remaining unlysed microbes, fresh resuspension buffer (800 μL), β-ME (5 μL) and 70 °C heated 10% SDS (200 μL) were sequentially added to the remaining pellet, mixed, homogenized and the supernatant was collected. The supernatants (lysates) from both homogenization steps of a sample were kept separate until nucleic acid pellets were dissolved and combined as described below.

The lysate was mixed with 200 μL of 10 M ammonium acetate, placed on ice for 5 min, and centrifuged at 4 °C for 10 min at 16,000 × g. The supernatant was mixed with an equal volume of isopropanol, placed on ice for 30 min, and centrifuged at 4 °C for 15 min at 16,000 × g. The nucleic acid pellet was washed with 70% ethanol and briefly dried. Pellets from all tubes corresponding to a sample were combined by dissolving in a total of 400 μL of TE [10 mM Tris.HCl pH 7.4; 1 mM EDTA].

To further purify metagenomic DNA, dissolved, pooled nucleic acids (400 μL) were mixed with 4 μL of DNase-free RNase (10 mg/mL) and incubated at 37 °C for 15 min. Subsequently, 30 μL of proteinase K (20 mg/mL) and 400 μL of Buffer AL (QIAamp DNA Stool Mini Kit; QIAGEN Inc. Toronto, ON, Canada) were added and the mixture was incubated at 70 °C for 10 min. Following incubation, absolute ethanol (400 μL) was added and a portion of the mixture (500 µL aliquot) was applied to the QIAamp column from the kit and centrifuged at 16,000 × g for 1 min. This step was repeated until all of the mixture corresponding to a sample was run through the column. The column was washed with AW1 and AW2 buffers as per manufacturer’s instructions and dried by centrifugation. To elute DNA, pre-warmed (70 °C) nuclease-free water (150 μL) was added to the column and held at room temperature for 2 min followed by centrifugation. The elution step was repeated with 100 μL of pre-warmed (70 °C) nuclease-free water yielding a total volume of ~250 μL of DNA.

To remove PCR-inhibitors, OneStep™ PCR Inhibitor Removal Kit (Zymo-Research Corp., Irvine, CA, USA) was used according to manufacturer’s instructions. Subsequent to DNA isolation, quality and quantity of the isolated DNA was evaluated. DNA concentrations were measured by fluorescence using the Quant-iT™ PicoGreen (Thermo Fisher Scientific, Mississauga,ON, Canada). Purity of the DNA was determined by measuring the ratios of absorbance at 260/280 and 260/230 using a NanoDrop spectrophotometer (Thermo Fisher Scientific). DNA preparations with a 260/280 ratio between 1.7–2.0 and a 260/230 ratio between 2.0–2.2 were regarded as pure. The presence of PCR-inhibitors was also evaluated by amplifying undiluted and various dilutions of a sample with universal 16S rRNA gene primers 27F and 1492R^39^.

### Metagenomic DNA sequencing and data processing

All library preparations, NGS and quality control steps were performed by the McGill University and Genome Quebec Innovation Centre, Montréal, QC, Canada. Illumina shotgun TruSeq libraries with insert size range 375–400 bp were prepared as per manufacturer’s instructions using Covaris-sheared metagenomics DNA and samples were run on an Illumina HiSeq. 2000 platform to generate 2 × 100 bp paired-end (PE) sequences. For the eight sample libraries, groups of four libraries each were multiplexed and run over two sequencing lanes per group in addition to all eight libraries being multiplexed in a single lane to obtain read data at various depths, as indicated in Fig. 1. As a quality control for cluster generation and sequencing, each HiSeq. 2000 sequencing lane was spiked with the PhiX174 *sensu lato* virus genomic DNA library at 1% concentration of the total DNA loaded per lane.

For each sample, to generate a FASTQ paired-end (PE) file dataset at a maximum available sequencing depth (D1), two corresponding FASTQ PE data file sets resulting from each of the duplicate D0.5 sequencing were combined (Fig. 1). As a result, 8 and 16 sets of FASTQ PE files for D1 and D0.5 depths respectively, were generated for eight samples. In addition, 8 sets of FASTQ PE data files originating from 8 samples multiplexed in a single sequencing lane constituted D0.25 data corresponding to 1/4^th^ depth compared to the largest grouping D1 (Fig. 1).

Trimmomatic^40^ was used to remove adapter contamination and low quality reads using the following parameters: trimming leading and the trailing low quality (quality score < 3) or ambiguous base calls from sequence reads; performing quality score filtering using a sliding window at every four bases with a minimum Phred score of 15; discarding sequences with <36 nucleotides; removing adapters supplied in the TruSeq. 3 adapter sequence file using a maximum of two mismatches in the initial seed, and clipping the adapter if a match score of 30 was reached. Singleton reads whereby the other pair was discarded were also included in downstream analysis.

### Microbiome and Resistome analyses

Data were analyzed within the Galaxy platform^41^, with a Galaxy Web server instance supported by the National Microbiology Laboratory, Public Health Agency of Canada (PHAC NML Galaxy). A custom workflow integrating Kraken taxonomic classification tools^20^ and the AmrPlusPlus resistome analysis pipeline tools^27^ was setup in Galaxy to obtain simultaneous outputs for both resistome and microbiome analyses of metagenomic sample reads from a single metagenome-resistome workflow. Following Trimmomatic as the first step in the workflow, Kraken and the AmrPlusPlus tools were programmed to run in parallel. For microbiome analysis, reads passing quality filters from Trimmomatic were classified using the updated custom Kraken database comprised of complete genomes in RefSeq for bacteria, viruses, fungi, protozoa and archaea (bvfpa). Kraken-filter script with a threshold of 0.05 was used in the classification pipeline to enhance the accuracy of taxonomic assignments. Reports of the taxonomic classification of reads were generated with Kraken’s report function. For the resistome analysis, the quality-filtered reads were provided as input to BWA-MEM alignment^42^ using default parameters including a mismatch penalty value of 4 to the MEGARes AMR genes database^27^, without the inclusion of host genome filtering step. Reads were assigned to ARGs using a 75% gene coverage/fraction threshold. Read counts originating from alignments to housekeeping genes associated with AMR (e.g., *rpoB*, *gyrA*, *parC*, etc.), present in the MEGARes database requiring single nucleotide polymorphism (SNP) confirmation, were filtered out from the AMR report before further analyses. Counts of short reads aligned to the ARGs were recorded and used for downstream comparative analyses.

### Microbiome and resistome comparison

For microbiome comparisons among datasets at various sequencing depths, reads for all eukaryotes and Enterobacteria phage PhiX174 *sensu lato* were filtered out from the Kraken reports with Pavian^43^. Complete taxonomic lineages were also added by Pavian. Sets of unique taxa at each sequencing depth were determined for each taxonomic level (i.e. phylum, class, family, order, genus, and species) and for each AMR classification level (i.e. class, mechanism, group, and gene) for non-rarefied data using a custom Python script using the *pandas* library (version 0.19.2; http://pandas.pydata.org/pandas-docs/version/0.19.2/), applying built-in set functions in Python version 3.4.2 (http://www.python.org), and plotting the output as Venn diagrams with the *matplotlib_venn package*^44^.

### Microbiome and resistome α-diversity and richness analyses

Species richness was calculated on non-rarified sample data using the ‘specnumber’ function of vegan. The distributions of species richness were visualized with box-and-whisker plots using the ggplot2 package of R^45^.

For α-diversity analysis, AMR and Kraken results were normalized at each sequencing depth using a data-driven approach based in shifts in the distributions of counts called Cumulative Sum Scaling (CSS) normalization^46^ of the metagenomeSeq package^47^ of R. Normalized counts were then aggregated at each of the taxonomic levels (phylum, class, order, family, genus, and species) and the antimicrobial resistance classification levels (class, mechanism, group, and gene). The Shannon and the Inverse Simpson indices of diversity^48^ were calculated on normalized counts for every sample with the diversity function of the vegan package of R. The distributions of indices at each sequencing depth were visualized as boxplots in the same manner as species richness.

### Microbiome and resistome rarefaction

A recently developed tool, Krakefaction (https://github.com/phac-nml/krakefaction), was used with Kraken assignment data to generate values for microbiome rarefaction curve. This software organizes sample reads into subsamples of regularly increasing sizes (5% increment for current study) and reports the number of taxa present within each subsample for all principal classification ranks. The rarefaction curves were plotted using the ggplot2 package of R as numbers of unique phyla, orders, genera and species as a function of sampling depth. For resistome, RarefactionAnalyzer tool of the AmrPlusPlus pipeline^27^ was used with 5% subsampling increments of the read data with 10 iterations at each level. The numbers of unique genes, mechanisms, and classes were then plotted as a function of sampling depth using the ggplot2 package of R.

### Statistical analysis

The Kruskal–Wallis test^49^ was performed to evaluate differences in richness and diversity for either ARGs or microbial taxa at various taxonomic ranks. Nemenyi post-hoc comparisons^50^ were conducted to determine statistical significance between two groups for incidences where differences were declared significant at P < 0.05 as per the Kruskal-Wallis analysis.

### Data Availability Statement

All Illumina sequence data from the current study have been deposited in NCBI’s Short Read Archive under BioProject ID PRJNA420682.

## Electronic supplementary material

Supplementary information

Dataset 1

Dataset 2

Dataset 3

Dataset 4

Dataset 5
