# Supplementary material for: Impact of sequencing depth on the characterization of the microbiome and resistome
Source: Sci Rep. 2018 Apr 12;8:5890. doi: 10.1038/s41598-018-24280-8 (PMC5897366; doi:10.1038/s41598-018-24280-8)
Supplement: Supplementary file 1 — Supplementary information [file 41598_2018_24280_MOESM1_ESM.pdf]

Impact of sequencing depth on the characterization of the microbiome and resistome.

Rahat Zaheer, Noelle Noyes, Rodrigo O. Polo, Shaun R. Cook, Eric Marinier, Gary Van Domselaar, Keith E. Belk, Paul S. Morley, Tim McAllister

Supplementary information (Table S1, Figures S1 – S6):

Table S1: DNA concentration (PicoGreen) and purity (NanoDrop) for samples submitted for sequencing.

Sample ID	Concentration (PicoGreen) (ng/µl)	Total quantity (µg)	260/280 ratio (NanoDrop)
N**003**	78.24	3.91	1.78
N**013**	50.69	2.53	1.82
S**034**	79.68	3.98	1.85
S**040**	59.00	2.95	1.84
V**046**	87.17	4.36	1.87
V**053**	89.09	4.45	1.87
A**062**	92.03	4.60	1.84
A**070**	104.26	5.21	1.84

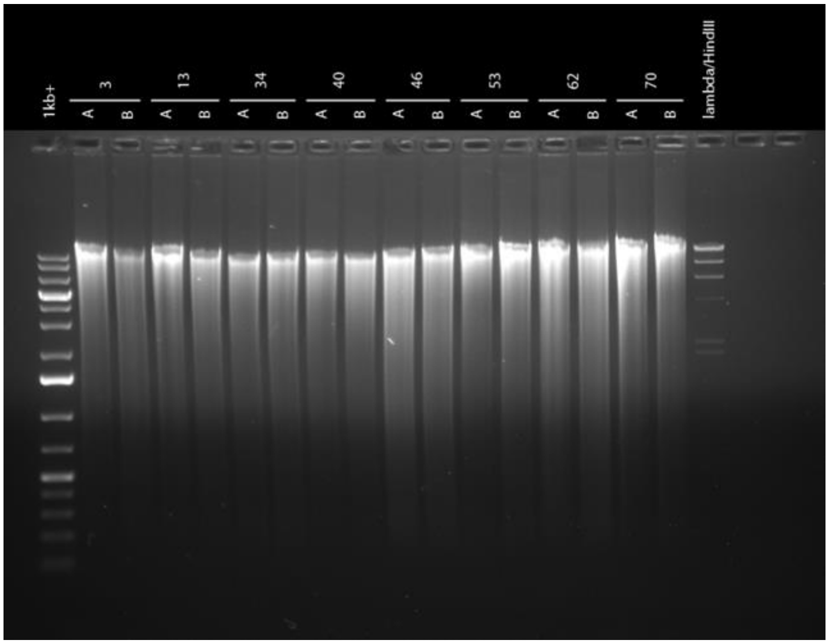

Figure S1: Gel electrophoresis of metagenomic DNA isolated. Loaded 4 µL on 1% agarose gel; run at 105V for 1 hr. Replicate samples A & B were pooled prior to submitting samples for sequencing.

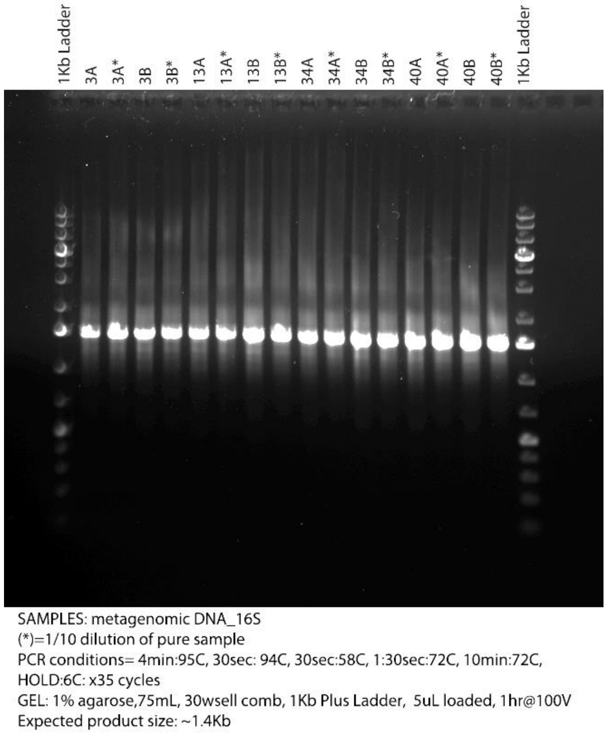

A

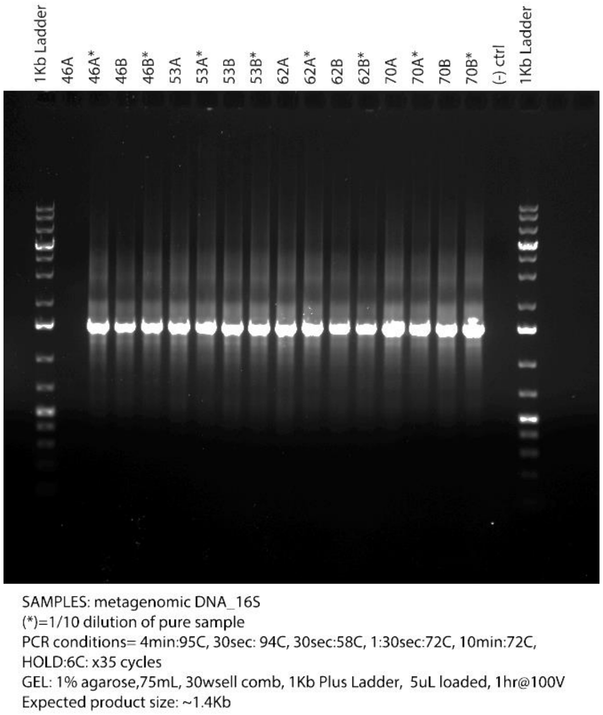

B

Figure S2 A & B: Control PCR targeting bacterial 16SrRNA gene (27F and 1492R primer pair). Both undiluted and diluted samples could be appropriately amplified indicating that DNA was free of PCR inhibitors.

NOTE: No sample was loaded in lane 46A in Figure S2 B.

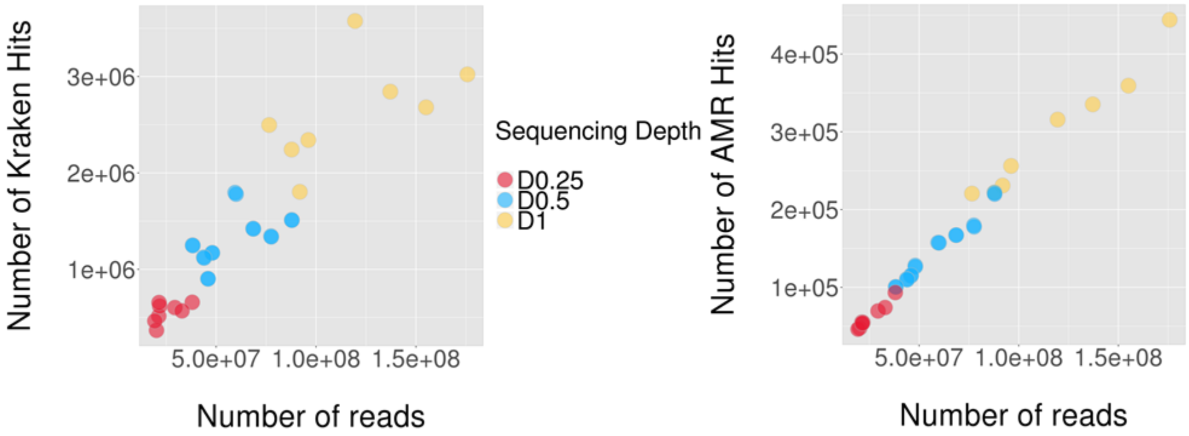

A **B**

Figure S3: Correlation between A) numbers of sequence reads and taxonomic assignments as identified by Kraken, and B) numbers of sequence reads and assignments to the MEGARes AMR database for D1 (n=8), D0.5 (n=2 x 8; 16 total) and D0.25 (n=8) samples. Values of duplicate samples in D0.5 dataset overlap, hence appearing as single dot.

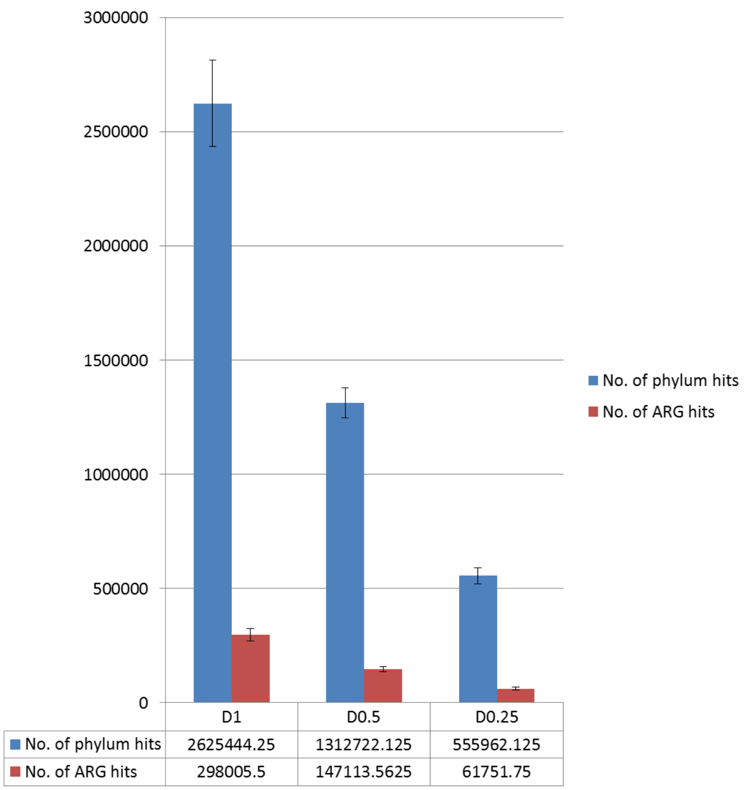

Figure S4: The average number of reads aligning to phyla and to the MEGARes AMR database at various sequencing depths increased on by ~2.4 fold for D0.5 compared to D0.25 and by ~2 fold for D1 compared to D0.5.

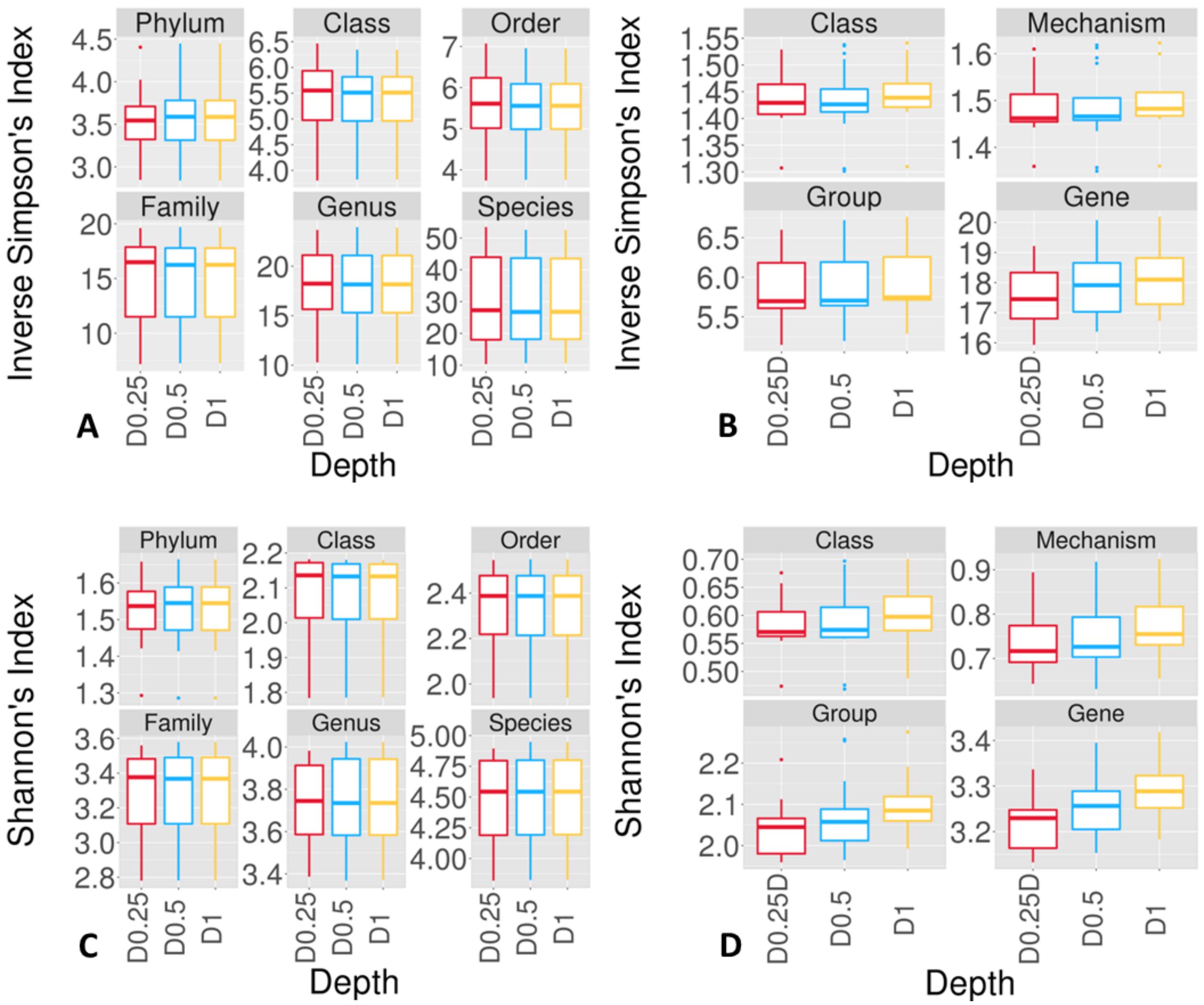

Figure S5: α-diversities of microbiome and resistome at various sequencing depths. Box-and-whisker plots showing A) microbial taxon α-diversity Inverse Simpson's index; B) AMR category α-diversity Inverse Simpson's index; C) microbial taxon α-diversity Shannon's index; D) AMR category α-diversity Shannon's index. Boxes represent the interquartile ranges (upper line is the 75% quantile, and the lower line is the 25% quantile), the lines inside the boxes are the medians, the whiskers span the range of the 25% quantile or the 75% quantile plus 1.5 times the interquartile range, and dots are outliers.

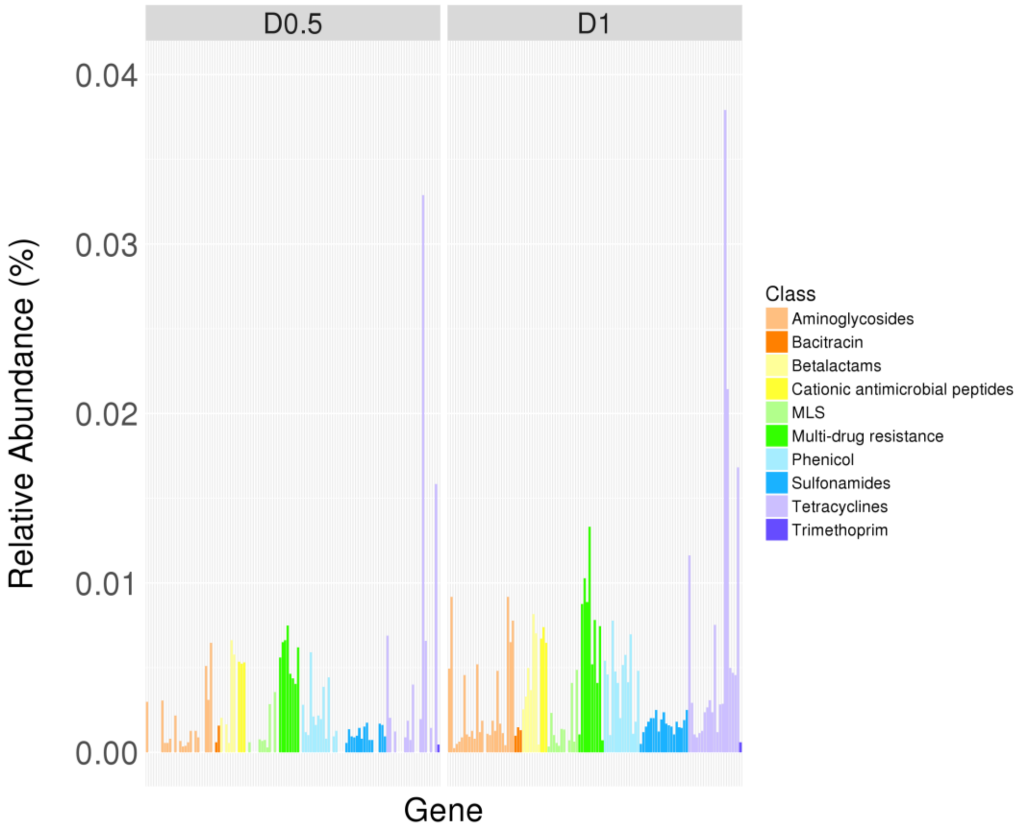

Figure S6: Relative abundance of ARGs missing in the D0.25 dataset but present in higher depth datasets D1 & D0.5.
